# Inverted S-Shaped Compact Antenna for X-Band Applications

**DOI:** 10.1155/2014/604375

**Published:** 2014-05-07

**Authors:** M. Samsuzzaman, M. T. Islam

**Affiliations:** Department of Electrical Electronic and Systems Engineering, Faculty of Engineering and Built Environment, Universiti Kebangsaan Malaysia, 43600 Bangi, Selangor, Malaysia

## Abstract

A novel probe-fed compact inverted S-shaped multifrequency patch antenna is designed. By employing two rectangular slots that change the conventional rectangular patch into an inverted S-shaped patch, the antenna is able to operate in triple frequency in the X-band. The performance criteria of the proposed design have been experimentally verified by fabricating a printed prototype. The measured results show that the −10 dB impedance bandwidth of the proposed antenna at lower band is 5.02% (8.69–9.14 GHz), at middle band is 9.13% (10.47–11.48 GHz), and at upper band is 3.79% (11.53–11.98 GHz). Two elliptical slots are introduced in the ground plane to increase the peak gain. The antenna is excited by a simple probe feeding mechanism. The overall antenna dimension is  0.52*λ* × 0.60*λ* × 0.046*λ* at a lower resonance frequency of 9.08 GHz. The antenna configuration and parametric investigation are conducted with the help of the high frequency structural simulator, and a good agreement is achieved between the simulated and measured data. The stable gain, omnidirectional radiation pattern, and consistent radiation efficiency in the achieved operating band make the proposed antenna a suitable candidate for X-band applications.

## 1. Introduction

Microstrip patch antenna implementation is a milestone in wireless communication systems and is continuing to fulfill the changing demands of the new generation of antenna technology. These antennas are widely used in present wireless communications systems because of their natural facilities of low profile, light weight, conformal design, low cost, ease of fabrication, and ease of integration with circuits. Since compact, low cost, multitasking, and reliable wireless communication devices have become an essential part of our daily communication life, the need for low profile miniaturized multi- and wideband antennas has escalated [1]. Communication systems that operate in the X-band are normally designed using separate antennas for each band. Because it is becoming more and more important to use such systems in one setting, it is desirable to design a single antenna that operates in multiple frequencies for multitasking.

Different types of multi- or wideband antennas have been improved to face the rising demands for a modern portable wireless communication device which is capable of integrating more than one communication standard into a single system. In recent years, many authors have dedicated their investigations to create new designs or variations in the original antenna that, to some extent, produce either wider bandwidths or multiple-frequency operation in a single element. Many techniques are implemented to achieve single- or multifrequency operations [2–24]. Multiband or wideband microstrip antenna was realized by cutting slots inside the patch [2–7] operation, again by applying fractal shape technique to antenna geometrics [8], using dielectric resonator [9, 10], using multilayer stacked patch [11–14], with shorted parasitic element [15], and single-layer microstrip antenna [16–24] has been paid much attention for achieving dual-band or multiband. Among the investigation, S-shaped antenna [2, 4–7, 11] has been reported for different applications. A microstrip S-shaped patch antenna was fed by a coaxial feeding, which was designed by inserting two slots into the rotated square patch, and it looked like the English letter “S” [2]. A 50 mm × 50 mm S-shaped meander patch antenna was designed for multiband applications [4]. A compact circularly polarized microstrip antenna with an S-shaped slot was proposed for dual-band operation but the antenna dimension was 0.458*λ* × 0.458*λ* × 0.095*λ* at 2.5 GHz [5]. The authors used two rectangular slots for creating resonance. Design of an S-shaped patch antenna fed by dual offset electromagnetically coupled to generate a 5-6 GHz frequency range was presented in [6]. An S-shaped patch antenna was designed for X-band applications where the authors introduced two slots to perturb the surface current path [7]. A printed S-shaped monopole antenna for pent band mobile phone application has been designed [11] where the antenna has a uniplanar structure and can be printed on an FR4 substrate of small size 10 mm × 45 mm patch but the ground plane is 45 mm × 100 mm. By mounting the antenna above the top edge of the system ground plane of the mobile phone and feeding it using a perpendicular feed, in this research, an inverted S-shaped triple frequency patch antenna has been proposed for X-band applications. So, some literatures have been studied specially for X-band applications [7, 9, 10, 13, 14, 17–24]. For X-band applications, an S-shaped patch antenna was designed where the authors introduced two slots to perturb the surface current path [7]. The antenna dimension was 30.08 mm × 45.9 mm and the antenna was working at 10 GHz. Microstrip-fed dielectric resonator antennas for X-band applications have been proposed in [9]. A slot antenna and a dielectric resonator antenna (DRA) were combined to effectively design a dual-band dielectric resonant antenna for C- and X-band applications, but this was achieved through a complex multilayered structure [10]. The axially fed microstrip X-band antenna consisting of a thin copper disk on the dielectric substrate with low permittivity has been presented [13]. The value of VSWR is less or equal to 2 in the working frequency band 8.46 to 10.1 GHz that corresponds to the antenna bandwidth of 18%. The proposed antenna was comprised of a high dielectric resonator, a microstrip-fed stepped patch, and an intermediate substrate. Although the antenna achieves wide bandwidth, it is costly and complex to design. An X-band woodpile electromagnetic bandgap (EBG) material has been designed for planar antenna to achieve high gain [14]. A slotted triangular-shaped C- and X-band patch antenna for satellite applications has been proposed with high dielectric and costly material [17]. A ceramic-polytetrafluoroethylene composite material-based miniaturized split-ring C- and X-band patch antenna was designed [18]. The proposed antenna obtained operating bandwidths (reflection coefficient <−10 dB) ranging from 5.0 to 6.5 GHz, 9.1 to 9.6 GHz, and 10.7 to 11 GHz. However, the antenna was designed on a high dielectric and costly substrate. A new configuration of patch antenna has been proposed [19] which consists of an S-band cross patch with four square patches printed in the empty spaces between the arms of the cross. These patches provide a subarray working at X-band. Here, dual offset guideline may lead to design complexities. A compact multi-T-shaped multiband monopole antenna with three inverted U-shaped slots has been proposed [20]. The proposed antenna operates at 2.25–2.7 GHz, 3.25–3.6 Hz, 4.95–6.2 GHz, and 7-8 GHz, covering the operation bands of Bluetooth, WiMAX,WLAN, and downlink of X-band satellite communication system, but the antenna dimension was 40 mm × 30 mm. An X-band directive single microstrip patch antenna has been proposed where used spaced superstrates were made from alumina sheets of two different sizes as dielectric parasites to increase the gain of the proposed antenna [21]. A broadband antenna operating in X-band applications was presented in [22]. The antenna is made of a polygon with a circular slot and a Teflon layer (Radom), which increases the performance of the antenna. A probe-fed rectangular microstrip antenna for X-band applications was presented in [23], but this was for only a single resonance frequency (9 GHz). A single feed compact circular microstrip antenna has been presented for X-band applications where L slits are introduced at the right edge of the patch to reduce the resonant frequency at 11.4 GHz. The slot increases the bandwidth up to only 210 MHz, with a return loss of −38.2 dB, absolute gain of approximately 4.82 dBi, and efficiency of the antenna of only 58.16%. In the literature review above, it can be easily concluded that the previously proposed designs are either high in design complexity, not miniaturized, costly, or lacking multiband support.

In this paper, a compact triple frequency slotted rectangular microstrip patch antenna consisting of two rectangular and a couple of twin embedded slots is presented. By selecting shapes and dimensions of these embedded slots properly, good multiband impedance bandwidths and suitable radiation characteristics for use in X-band (8–12 GHz) applications could be obtained. The effects of the embedded slots on the resonance were studied. The proposed antenna not only has good multiband operation performance and radiation pattern but also a simple structure and compact size. The details of the antenna design and experimental results are presented in the following sections.

## 2. Antenna Design, Architecture, and Optimization

**Figure 1:**
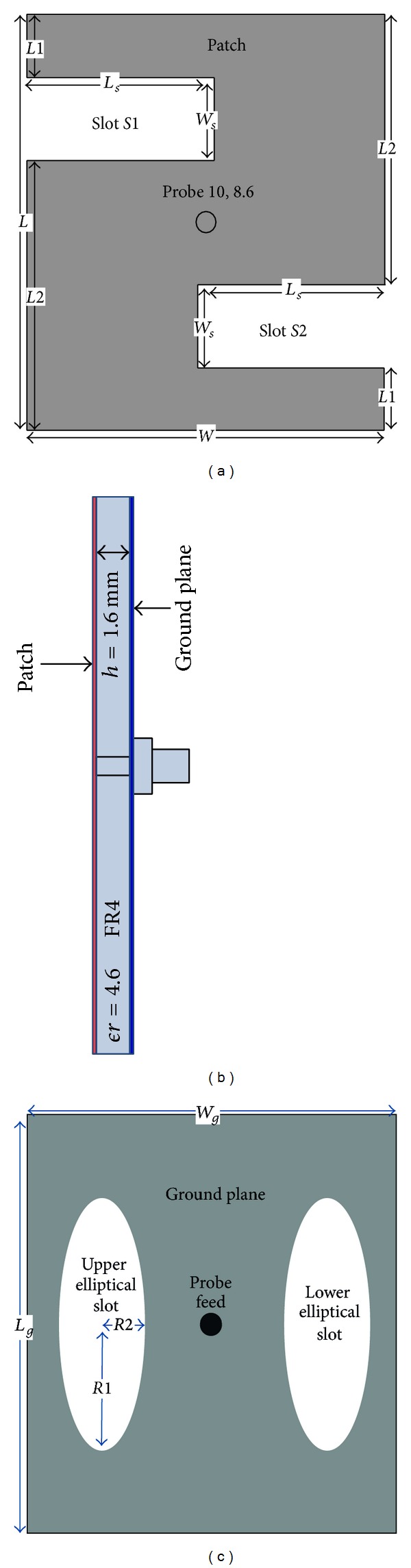
Proposed antenna geometry: (a) top view, (b) side view, and (c) bottom view.

The geometry and configuration of the proposed microstrip antenna are illustrated in Figure 1. Firstly, a rectangular microstrip patch antenna is designed to resolve the length (*L*) and width (*W*) following the rules of standard design procedure. Then, two rectangular slots are organized to agitate the surface current path, initiating a local inductive effect that is responsible for resonance in the antenna, which constitutes the inverted S-shaped patch. The two slots in the rectangular patch can reduce the area of the patch. This means that the space required for antenna fabrication is less than the conventional rectangular patch antenna dedicated for wideband operation usage at a fixed operating frequency. The slot length (*L*_*s*_) and slot width (*W*_*s*_) of the patch regulate the frequency of the fundamental resonant mode. The dimensions of slots, that is, width and length, always affect the performance of the antenna as discussed. The slot dimensions of the antenna are *L*_*s*_ = 9.0 mm and *W*_*s*_ = 4.0 mm. Effects of slots on the performance of the antenna can be measured by modeling the antenna in terms of its inductance, capacitance, and load resistance. The dimensions of the patch of the antenna for the resonant frequency are calculated to be *L* = 20.0 mm and *W* = 17.2 mm using standard design equations for rectangular microstrip antenna design. The substrate is taken as FR4 having relative permittivity equal to 4.6 and thickness equal to 1.6 mm. Two slots with elliptical shapes are embedded in the ground in the opposite position to determine the major radius (*R*1) and ratio (*R*2). The location (10, 8.6) of the coaxial probe-feed is also shown in Figure 1(a) as well.

## 3. Results and Analysis

In this section, different types of parametric study of the proposed antenna are carried out and presented. For the parametric studies, the numerical simulations have been performed by the finite element method- (FEM-) based commercial simulator HFSS 15.0. To improve the antenna characteristics such as bandwidth, gain, and return loss performance of the proposed antenna, several parameters of the antenna have been investigated. Optimal parameter values of the antenna are listed in Table 1.

**Figure 2:**
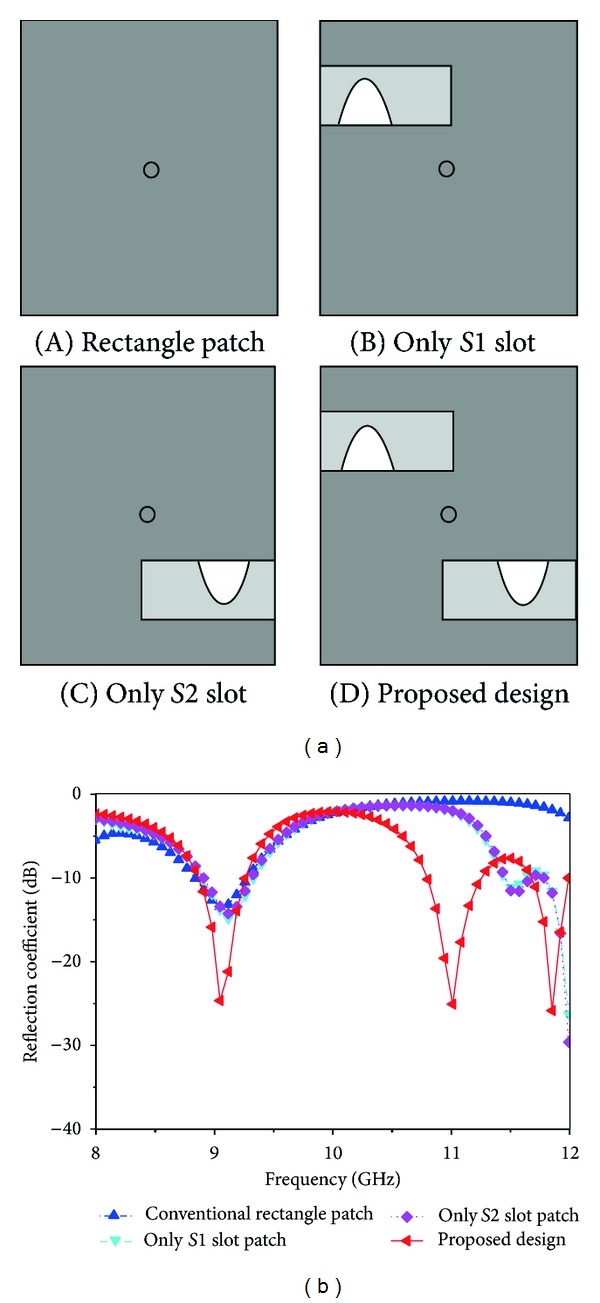
(a) Proposed antenna design evolution and (b) simulated reflection coefficient for different patch shapes.

Design evaluation of proposed patch is depicted in Figure 2(a). The simulated reflection coefficients of the conventional rectangular patch, with only the *S*1 slot, only the *S*2 slot, and the proposed design, are depicted in Figure 2(b). From this Figure 2(b), it can be easily noted that, by using a conventional rectangular patch, there is only one resonance frequency, which is 9.07 GHz, and return loss is −13.53 dB with an impedance bandwidth of 440 MHz. However, by cutting the *S*1 and *S*2 slots in that rectangular patch, there is still a single resonance frequency, 9.13 GHz and 9.12 GHz, and return loss, −14.92 dB and −14.33 dB, for each slot. From Figure 2, it can be concluded that for the conventional rectangular patch, etching *S*1 and *S*2 slots, there is no higher resonance frequency. Alternately, due to the combined presence of the *S*1 and *S*2 slots, multifrequency operations are obtained with large values of the frequency impedance bandwidth. For the proposed inverted S-shaped antenna, return loss is −27.78 dB at 9.08 GHz, −25.61 dB at 11.00 GHz, and −26.98 dB at 11.86 GHz and the corresponding bandwidth is 390 MHz, 450 MHz, and 310 MHz, respectively.

### 3.1. Effect of Parameters *L*_*s*_ and *W*_*s*_ of the Proposed Antenna

**Figure 3:**
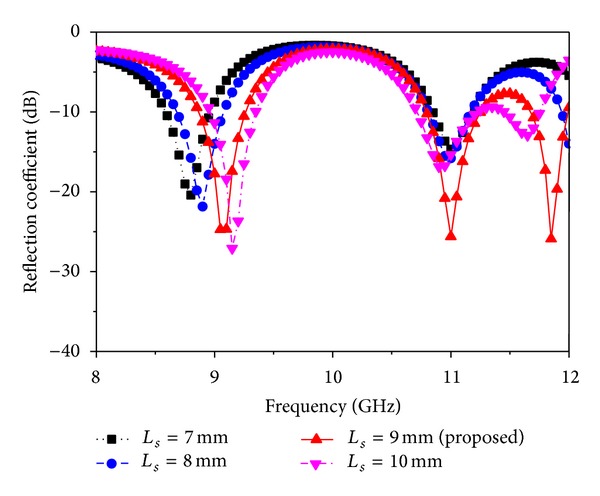
Simulated reflection coefficient for different values of *L*_*s*_ (other parameters: *L* = 20 mm, *W* = 17.2 mm, *L*1 = 3 mm, *L*2 = 13 mm, *W*_*s*_ = 4 mm, *L*_*g*_ = 20 mm, *W*_*g*_ = 17.2 mm, *R*1 = 2 mm, *R*2 = 3 mm, *X*_*f*_, *Y*_*f*_ = 10, 8.6).

**Figure 4:**
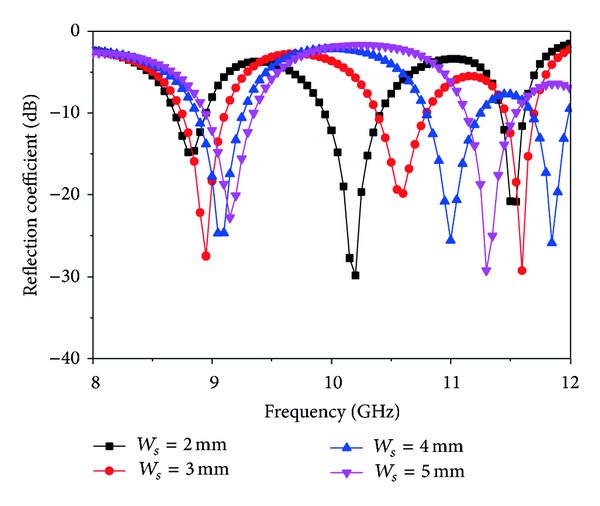
Simulated reflection coefficient for different values of *W*_*s*_ (other parameters: *L* = 20 mm, *W* = 17.2 mm, *L*1 = 3 mm, *L*2 = 13 mm, *L*_*s*_ = 9 mm, *L*_*g*_ = 20 mm, *W*_*g*_ = 17.2 mm, *R*1 = 2 mm, *R*2 = 3 mm, *X*_*f*_, *Y*_*f*_ = 10, 8.6).

For the fixed values of *W*_*s*_, *L*1, *L*2, *R*1, and *R*2 and the feed position (10, 8.6) of the patch, *L*_*s*_ is varied, and simulation results are depicted in Figure 3. If the values of *L*_*s*_ are increased above 9 mm, the resonance frequency is shifted for the first value, and the returning loss curve goes above the −10 dB line and the bandwidth is significantly decreased for the others. For *L*_*s*_ values less than 9 mm, the frequency is shifted to a lower value for the first, the same for the second, and the third one has no resonance. As the resonant frequency shifts towards a lower value, size reduction of the antenna is also reduced. Again, for the fixed values of *L*_*s*_, *L*1, *R*1, *R*2, and the feed position (10, 8.6) of the patch, the width, *W*_*s*_, of slots *S*1 and *S*2 is varied and displayed in Figure 4. If the values of *W*_*s*_ are less than 4 mm, then bandwidth and return loss are decreased. When stubwidth, *W*_*s*_, is increased to more than 4 mm, the resonance is shifted higher, and only two are lower than the −10 dB line; the size reduction of the antenna is also reduced with the reduction of the operating bandwidth.

### 3.2. Effect of Parameters *L*1 and *L*2 and Feed Position of the Proposed Antenna

**Figure 5:**
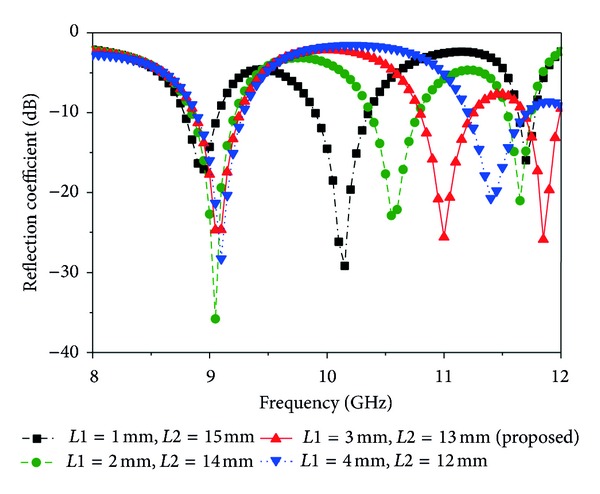
Simulated reflection coefficient for different values of *L*1 and *L*2 (other parameters: *L* = 20 mm, *W* = 17.2 mm, *L*_*s*_ = 9 mm, *W*_*s*_ = 4 mm, *L*_*g*_ = 20 mm, *W*_*g*_ = 17.2 mm, *R*1 = 2 mm, *R*2 = 3 mm, *X*_*f*_, *Y*_*f*_ = 10, 8.6).

For the fixed values of *L*_*s*_, *W*_*s*_, *R*1, and *R*2 and the feed position (10, 8.6), the lengths, *L*1 and *L*2, of both sides of the rectangular patch are varied and simulation results are displayed in Figure 5. If *L*1 is less than 3 mm and *L*2 is greater than 13 mm, return loss and bandwidth are decreased. Alternately, if *L*1 is greater than 3 mm and *L*2 is less than *L*3 mm, then the third frequency is completely missing.

**Figure 6:**
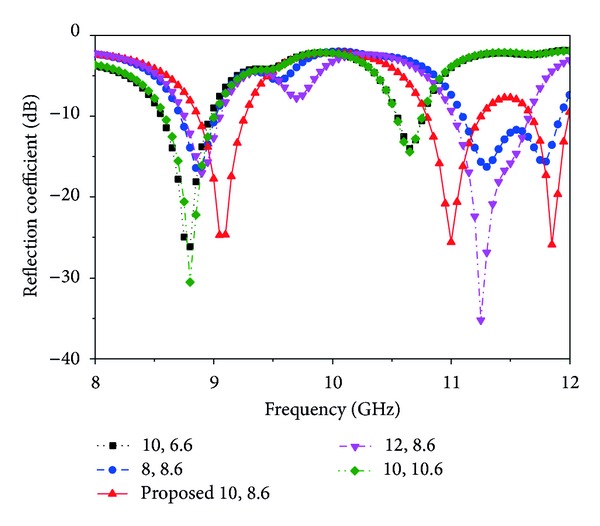
Simulated reflection coefficient for different feed positions (other parameters: *L* = 20 mm, *W* = 17.2 mm, *L*1 = 3 mm, *L*2 = 13 mm, *L*_*s*_ = 9 mm, *W*_*s*_ = 4 mm, *L*_*g*_ = 20 mm, *W*_*g*_ = 17.2 mm, *R*1 = 2 mm, *R*2 = 3 mm, *X*_*f*_, *Y*_*f*_ = 10, 8.6).

The feeding position is an important issue to be considered when designing a microstrip patch antenna. With the selection of the proper position, impedance matching can be obtained and, thus, the desired resonance.  Figure 6 shows the return loss graph obtained from the five different feed positions of the patch antenna. Due to the variation of excitation position, different return loss curves are obtained, and, in some cases, no resonance occurs. The positions are oriented around the patch. The best position is found at (8.6, 10), in the middle of the two slots, where the minimum return loss values are −27.78 dB at 9.08 GHz, −25.61 dB at 11.0 GHz, and −26.98 dB at 11.86 GHz.

### 3.3. Effect of Ground Plane Modification

**Figure 7:**
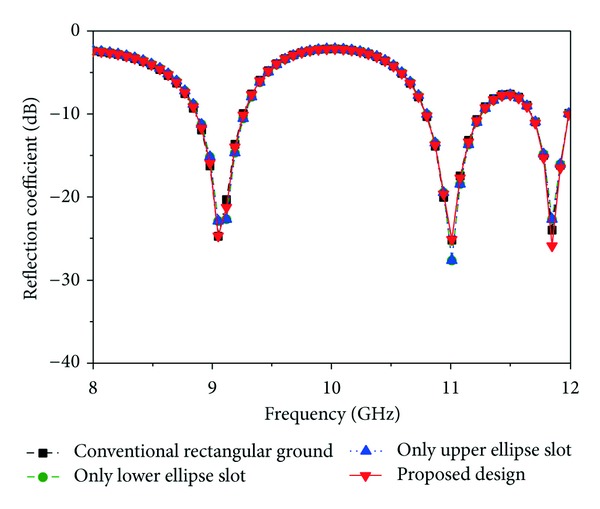
Simulated reflection coefficient for the effect of ground plane modification (other parameters: *L* = 20 mm, *W* = 17.2 mm, *L*1 = 3 mm, *L*2 = 13 mm, *L*_*s*_ = 9 mm, *W*_*s*_ = 4 mm, *L*_*g*_ = 20 mm, *W*_*g*_ = 17.2 mm, *R*1 = 2 mm, *R*2 = 3 mm, *X*_*f*_, *Y*_*f*_ = 10, 8.6).

**Figure 8:**
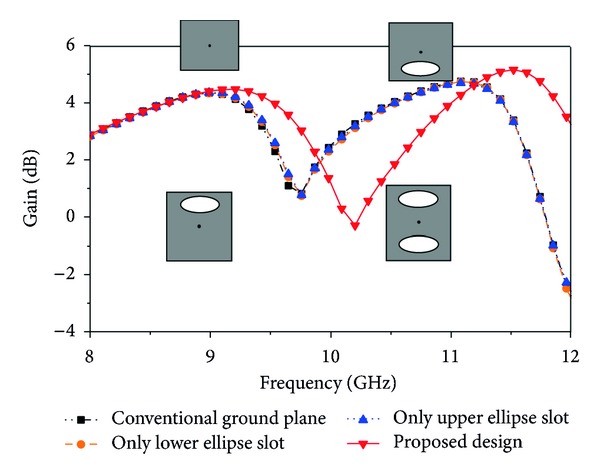
Simulated peak gain for the effect of ground plane modification (other parameters: *L* = 20 mm, *W* = 17.2 mm, *L*1 = 3 mm, *L*2 = 13 mm, *L*_*s*_ = 9 mm, *W*_*s*_ = 4 mm, *L*_*g*_ = 20 mm, *W*_*g*_ = 17.2 mm, *R*1 = 2 mm, *R*2 = 3 mm, *X*_*f*_, *Y*_*f*_ = 10, 8.6).

For the fixed values of *L*_*s*_, *W*_*s*_, *R*1, and *R*2 and the feed position (10, 8.6), *L*1 and *L*2, the rectangular ground plane, are modified by slot and displayed the return loss and peak gain results in Figures 7 and 8. From Figure 7, it can be observed that there is no effect on return loss, but, from Figure 8, there is an effect on peak gain. The gain is compared with four design modifications in the ground plane. A conventional ground plane, only the lower ellipse slot, only the upper ellipse slot, and, finally, the proposed design have been mentioned in Figure 8. The proposed ground plane has introduced a significant improvement of the gain in the achieved operating band.

### 3.4. Radiation Efficiency and Input Impedance

**Figure 9:**
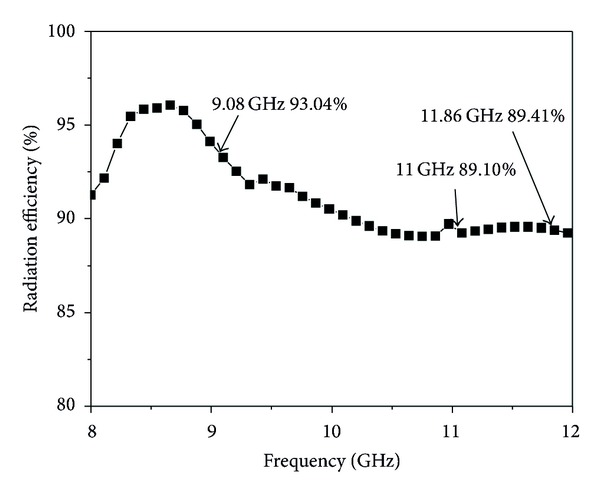
Radiation efficiency of the proposed antenna.

**Figure 10:**
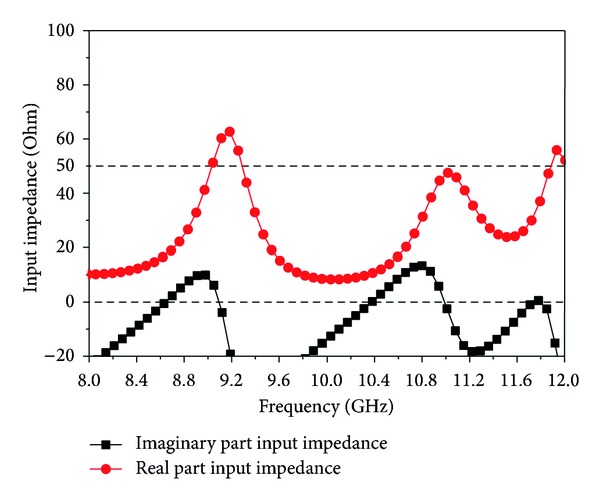
Input impedance of the proposed antenna.

The radiation efficiency of the proposed antenna is illustrated in Figure 9. It is observed that the designed antenna achieved a maximum radiation efficiency, at three bands, of 90.90%, 87.89%, and 88.32%, respectively. The input impedance of the proposed antenna is shown in Figure 10. The real part of the impedance has attempted to be as close as possible to 50 Ohms in the desired three bands. On the other hand, the imaginary part of the impedance has tried to be as close as possible to zero.

**Figure 11:**
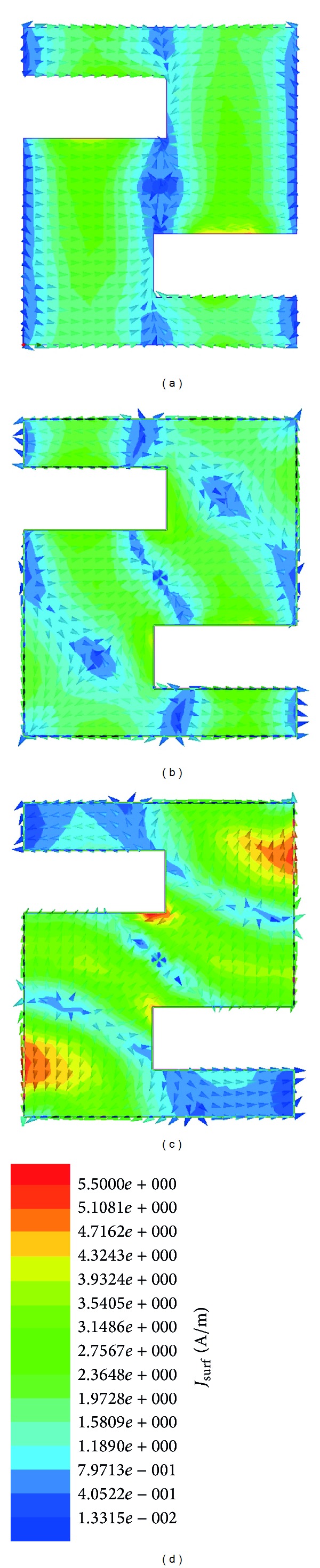
Current distribution at (a) 9.08 GHz, (b) 11.00 GHz, (c) 11.86 GHz, and (d) scale.

Figure 11 clearly stated the surface current distribution at three resonance frequencies of 9.08, 11, and 11.86 GHz, respectively. Through a numerical study of the vector and magnitude surface current distribution of the antenna, three characteristics current modes were found to exist in an achieved operating band. Each current mode was dominant at each resonance of 9.08, 11, and 11.86 GHz, respectively. It can be seen for three resonance frequencies that the edge of active two slots close to the feed point is showing the most current distribution, which amounts to   4.44 Am^−1^ for 9.08 GHz, 5.50 Am^−1^ for 11 GHz, and 4.44 Am^−1  ^ for 11.86 GHz. It is due to the coupling between the slot edges. Thus, at the lower frequency band, the current intensity is much weaker than that at higher frequencies. At the low frequency of 9.08 GHz, Figure 11(a) shows the current is evenly distributed on the patch radiator but mostly current is concentrate on the left and right rectangle and null point is created around the feed point. At a higher frequency of 11 GHz, the current shown in Figure 11(b) is still roughly symmetric distributed on the radiator. At the higher frequency of 11.86 GHz, higher order current modes are excited and the current density is no longer symmetrically distributed on the radiator as shown in Figure 11(c).

### 3.5. Prototyping and Measurement

**Figure 12:**
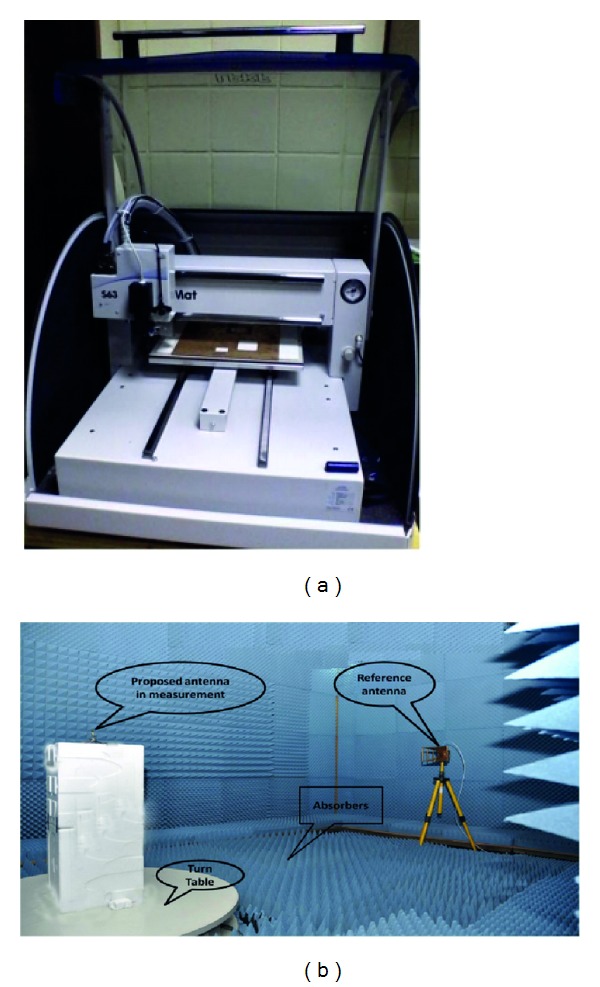
(a) LPKF machine (S63) and (b) anechoic chamber.

**Figure 13:**
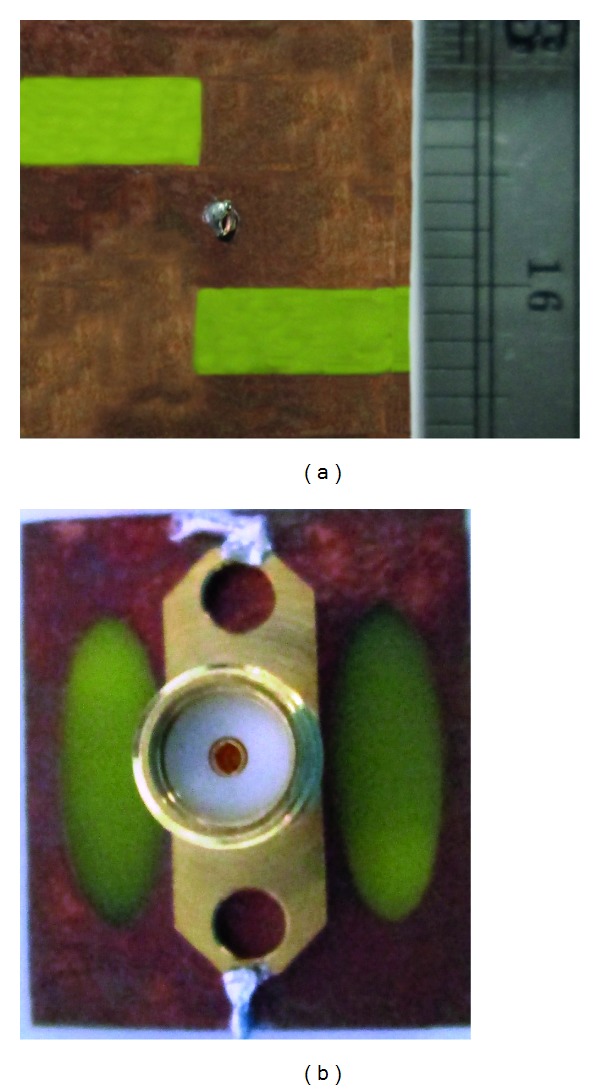
Proposed antenna prototype: (a) top view and (b) bottom view.

**Figure 14:**
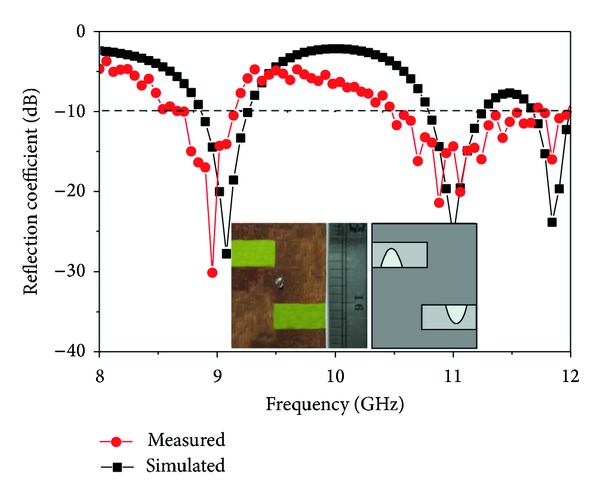
Comparison between measured and simulated reflection coefficient.

The performance characteristics of the proposed inverted S-shaped antenna have been analyzed, investigated, and optimized by utilizing the 3D electromagnetic structure solving functionality of the ANSYS' FEM-based high frequency structural simulator (HFSS). The accomplishment of the parametric studies gives an optimized geometric structure of the proposed inverted S-shaped antenna with defected ground plane, which is prototyped in the PCB LPKF (S63) prototyping machine to obtain a physical test model, which is shown in Figure 12(a). An anechoic chamber is the most widely used electromagnetic measurement system. The results of the proposed antenna prototype have been measured in a rectangular-shaped 5.5 m × 5 m × 3.5 m anechoic measurement chamber. A double ridge guide horn antenna has been used as a reference antenna. The measuring antenna is placed face-to-face with the reference antenna. The photograph of the anechoic measurement chamber is shown in Figure 12(b). A pyramidal-shaped electrically thick foam absorber has been used on the wall, ceiling, and floor with less than −60 dB reflectivity at normal incidence. A turn table of 1.2 m diameter has been used to rotate the measuring antenna with the specification, 1 RPM rotation speed; 360° rotation angle was connected with a 10 meter cable between controllers. An Agilent vector network analyzer (VNA E8362C) that ranges up to 20 GHz has been used for the measurement procedure. The prototype of the proposed antenna was fabricated and tested, which is portrayed in Figures 13 and 14. The comparison of the measured return loss with the simulated return loss is shown in Figure 14. The discrepancy between the simulated and measured results is due to the effect of improper soldering of the SMA connector or fabrication tolerance. However, at the first resonance, the bandwidth is 450 MHz and starts from 8.69 GHz to 9.14 GHz, which is a 140 MHz increment from the simulated result. Again at the second resonance frequency, the bandwidth of the measured result is 1010 MHz and starts from 10.47 GHz up to 11.48 GHz, which is a 560 MHz increment from the simulated impedance bandwidth. Finally, the third impedance bandwidth can be found at 450 MHz, starting at 11.53 GHz, and ending at 11.98 GHz.

**Figure 15:**
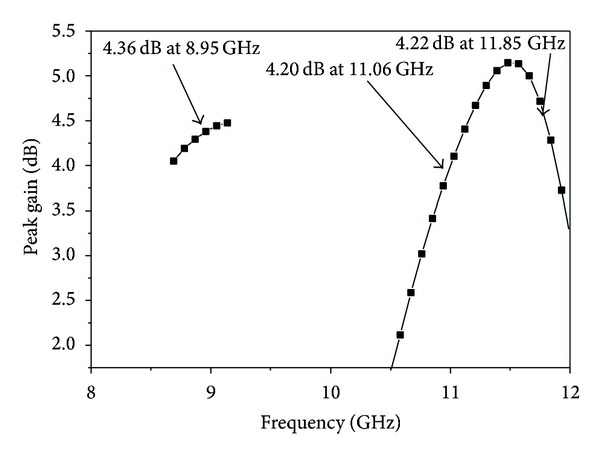
Peak gain of the proposed antenna.

Free-space ranges are used to measure the gain of the designed triple frequency X-band antenna by utilizing two identical horn antennas whose gain and radiation patterns are known.  Figure 15 shows the peak gain versus frequency plot for the proposed shape antenna. It is narrated that the first frequency bandwidth is approximately 450 MHz (8.69–9.14 GHz), the average peak gain for that operating band is 4.45 dB, the second frequency band is 1010 MHz (10.47–11.48 GHz), the average peak gain for that operating band is 3.99 dB, the last frequency band is 450 MHz (11.53–11.98 GHz), and the average peak gain for that operating band is 4.17 dB.

**Figure 16:**
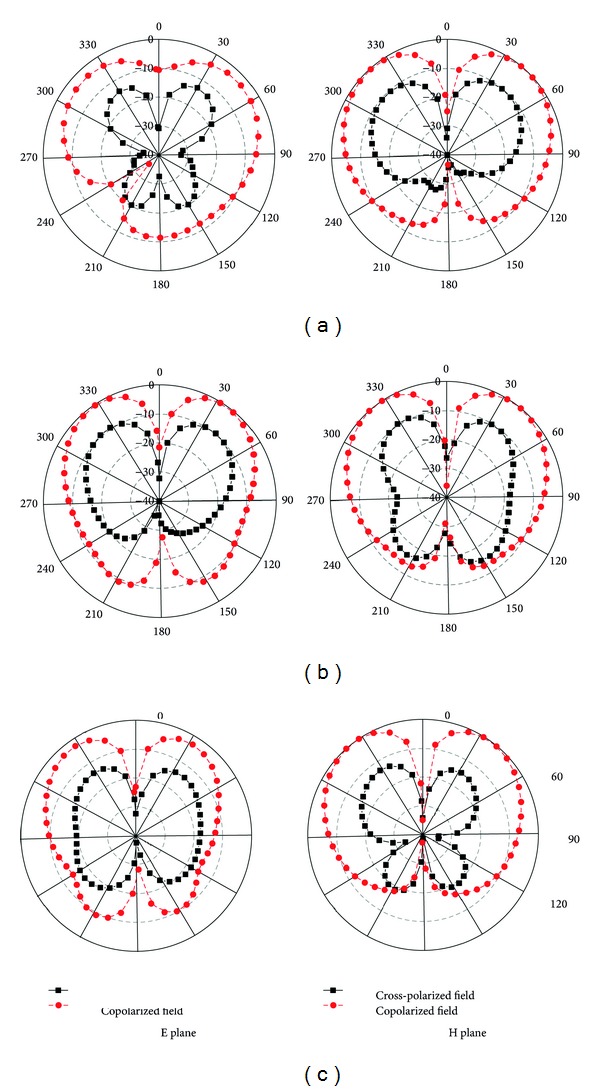
Radiation pattern at (a) 8.95 GHz, (b) 11.06 GHz, and (c) 11.85 GHz.

E and H plane radiation patterns for the proposed antenna at the three resonance frequencies are shown in Figure 16. It is observed that the copolarization is nearly donut-shaped and the direction of the proposed antenna is an omnidirectional radiation pattern. The cross polarization is low compared to copolarization.

The proposed antenna characteristics are compared with some existing antennas in Table 2. It can be easily stated that the reported antennas are larger in size, lower in gain, or less efficient compared to the proposed antenna.

## 4. Conclusion

In this study, an inverted S-shaped, compact, low profile multifrequency patch antenna is designed to obtain the desired resonant frequencies for X-band applications. The proposed antenna is composed of two rectangular slots in the patch, which make it like an inverted S-Shape. Two elliptical slots in the ground plane have been used to increase the gain. To validate this design concept, an antenna prototype has been fabricated and measured. Simulated and measured results show that the two rectangular slots reduced the size of the proposed antenna by 20.63% and also obtained the three resonance frequencies with higher bandwidth and stable gain. Antenna radiation efficiency has been achieved at approximately 95.10–92.20% for the first frequency band, 89.07–89.40% for the second frequency band, and 89.59–89.01% for the third frequency band operation. An optimization between bandwidth enhancement and size reduction is also maintained in this research. With these features, this antenna can be used in wireless communication systems operating at X-band.

## Figures and Tables

**Table 1:** Optimized antenna parameter (mm).

*L*	*W*	*L*1	*L*2	*L*_*s*_	*W*_*s*_	*L*_*g*_	*W*_*g*_	*R*1	*R*2
**20**	17.2	3	13	9	4	20	17.2	2	3

**Table 2:** Comparison between the proposed and some existing antennas.

Article	Dimension (mm × mm)	Resonance frequency (GHz)	Bandwidth (MHz)	Average gain (dB)
[9]	43.25 × 30	10.16	3700	N/A
[7]	30.08 × 45.9	10	1100	6
[21]	25.4 × 25.4	9.76	N/A	N/A
[22]	17.56 × 18.04	9.75	1801	3.56
[23]	11.01 × 13.9	9	110	N/A
[24]	4 × 9	11.4	210	4.82
Proposed	20 × 17.2	8.95, 11.06, 11.85	450, 1010, 450	4.45, 3.99, 4.17
